# Sex modulates the association of radial artery augmentation index with renal function decline in individuals without chronic kidney disease

**DOI:** 10.1007/s11255-020-02776-5

**Published:** 2021-01-12

**Authors:** Qiao Qin, Fangfang Fan, Jia Jia, Yan Zhang, Bo Zheng

**Affiliations:** 1 Department of Cardiology, Institute of Cardiovascular Disease, Peking University First Hospital, No. 8 Xishiku Street, Xicheng District, Beijing, 100034 China; 2 Institute of Cardiovascular Disease, Peking University First Hospital, Beijing, 100034 China

**Keywords:** Arterial stiffness, Radial augmentation index, Renal function decline, Sex

## Abstract

**Purpose:**

An increase in arterial stiffness is associated with rapid renal function decline (RFD) in patients with chronic kidney disease (CKD). The aim of this study was to investigate whether the radial augmentation index (rAI), a surrogate marker of arterial stiffness, affects RFD in individuals without CKD.

**Methods:**

A total of 3165 Chinese participants from an atherosclerosis cohort with estimated glomerular filtration rates (eGFR) of ≥ 60 mL/min/1.73 m^2^ were included in this study. The baseline rAI normalized to a heart rate of 75 beats/min (rAIp75) was obtained using an arterial applanation tonometry probe. The eGFRs at both baseline and follow-up were calculated using the equation derived from the Chronic Kidney Disease Epidemiology Collaboration. The association of the rAIp75 with RFD (defined as a drop in the eGFR category accompanied by a ≥ 25% drop in eGFR from baseline or a sustained decline in eGFR of > 5 mL/min/1.73 m^2^/year) was evaluated using the multivariate regression model.

**Results:**

During the 2.35-year follow-up, the incidence of RFD was 7.30%. The rAIp75 had no statistically independent association with RFD after adjustment for possible confounders (adjusted odds ratio = 1.12, 95% confidence interval: 0.99–1.27, *p* = 0.074). When stratified according to sex, the rAIp75 was significantly associated with RFD in women, but not in men (adjusted odds ratio and 95% confidence interval: 1.23[1.06–1.43], *p* = 0.007 for women, 0.94[0.76–1.16], *p* = 0.542 for men; *p* for interaction = 0.038).

**Conclusion:**

The rAI might help screen for those at high risk of early rapid RFD in women without CKD.

## Introduction

Chronic kidney disease (CKD) is increasingly recognized as a global public health problem, which affects 7–12% of the adult population in different regions of the world. Patients with CKD have a significantly increased risk of death, mainly due to cardiovascular disease that is followed by end-stage renal disease [1, 2]. Therefore, it is crucial to identify those at risk of fast renal function decline (RFD) amongst individuals without CKD as a primary preventive strategy.

Unlike the vascular beds of other organs, renal microcirculation exhibits low impedance and high flow, which make it vulnerable to pulsatile changes in blood flow. With increased arterial stiffness, the cushioning function of the arterial system, which converts the pulsed blood flow from the heart to the continuous blood flow of the capillaries, decreases [3, 4]. Renal microcirculation is exposed to high-pressure pulsatility, which may result in glomerular endothelial dysfunction and decreased renal arterial volume [5, 6].

Previous observational studies have provided solid evidence that arterial stiffness is associated with a faster decline in the estimated glomerular filtration rate (eGFR) in CKD patients [7–10]. However, there is no consensus yet on whether an association between arterial stiffness and RFD exists in individuals with preserved renal function [5, 11–14].

The radial augmentation index (rAI), a surrogate marker of arterial stiffness, is obtained by an automated tonometer against the radial artery in a seated position [15, 16]. The rAI is easily measured and is thus considered a suitable marker of arterial stiffness and its association with RFD in a large population with preserved renal function [17]. As the relationship between the rAI and renal function in individuals without CKD is not well understood, we examined this relationship in a Chinese community population with an eGFR of ≥ 60 mL/min/1.73 m^2^.

## Methods

### Study population

We used data from an atherosclerosis cohort from December 2011 to July 2014 in the Gucheng and Pingguoyuan communities of the Shijingshan district in Beijing, China. Detailed study procedures have been described previously [18]. In brief, a baseline survey was collected for 5962 residents aged ≥ 40 years between December 2011 and April 2012, and 3823 (64.1%) residents returned for a follow-up visit from May to July 2014. The difference in baseline characteristics was not statistically significant between the residents that returned for a follow-up and those that did not. We excluded participants without serum creatinine measurements upon follow-up who already had CKD defined by an eGFR of < 60 mL/min/1.73 m^2^ at baseline and who did not have a rAI measurement at baseline. Finally, 3165 participants were included in this study. Informed consent was obtained from all participants, and the study protocol was approved by the ethics committee of Peking University First Hospital.

### Data collection

Baseline data were obtained by trained research staff according to a standard operating procedure. All participants were interviewed using a standardized questionnaire, including demographic characteristics, health behavior, and medical history. Anthropometric measurements were taken according to a standard operating procedure.

After a 5-min rest, seated brachial blood pressure (BP) and pulse measurements were obtained from each participant using an Omron HEM-7117 electronic sphygmomanometer (Omron Health Care, Kyoto, Japan). Triplicate measurements on the right arm were taken with ≥ 1 min between successive readings. Systolic BP (SBP), diastolic BP (DBP), and pulse were averaged over three consecutive measurements for each participant.

To assess arterial stiffness, the rAI was obtained from the radial arterial waveform by an arterial applanation tonometry probe (HEM-9000AI; Omron Healthcare, Kyoto, Japan). The first and second peaks of brachial systolic pressure (SP1 and SP2) and brachial diastolic pressure were automatically determined using the fourth derivatives for each radial arterial waveform. The rAI was calculated as follows: (SP2−brachial DBP)/(SP1−brachial DBP) × 100 (%). Since the rAI is influenced by heart rate, it was normalized to 75 beats/min (rAIp75) according to previous guidelines [19].

Venous blood samples were obtained from the forearm of participants that had fasted overnight. Fasting blood glucose, 2 h glucose concentration in the standard 75 g oral glucose tolerance test, total cholesterol, low-density lipoprotein cholesterol, high-density lipoprotein cholesterol, and triglyceride concentrations were measured using a Roche C8000 Automatic Analyzer. As described previously [18], serum creatinine concentration at baseline and follow-up were analyzed using the enzymatic method and Jaffe’s kinetic method, respectively, in different laboratory centers. To ensure comparability, the serum creatinine concentrations at baseline and follow-up were transformed into values by the enzymatic method and finally calibrated to the values of one core laboratory. The eGFR was calculated using the equation published by the Chronic Kidney Disease Epidemiology Collaboration [20].

### Outcome

The outcome assessed in this study was RFD during the follow-up period, defined according to the Kidney Disease: Improving Global Outcome 2012 definition as follows: a drop in the GFR category (≥ 90 [G1]), 60–89 [G2], 45–59 [G3a], 30–44 [G3b], 15–29 [G4], and < 15 [G5] mL/min/1.73 m^2^) accompanied by a ≥ 25% drop in eGFR from baseline or a sustained decline in eGFR of > 5 mL/min/1.73 m^2^/year [21].

### Definitions

Current smoking was defined as smoking at least one cigarette per day for at least half a year. Current drinking was defined as drinking alcohol at least once per week for at least half a year. Body mass index (BMI) was calculated as weight in kilograms divided by the square of height in meters. Hypertension at baseline was defined as any self-reported history of hypertension, or a SBP ≥ 140 mmHg or DBP ≥ 90 mmHg, or prescribed antihypertensive drugs. Diabetes mellitus at baseline was defined as any self-reported history of diabetes, fasting blood glucose levels ≥ 7.0 mmol/L, oral glucose tolerance test ≥ 11.1 mmol/L, or prescribed hypoglycemic drugs. Dyslipidemia at baseline was defined as any self-reported history of hyperlipidemia, total cholesterol levels ≥ 5.18 mmol/L (200 mg/dL), low-density lipoprotein cholesterol levels > 3.37 mmol/L (130 mg/dL), high-density lipoprotein cholesterol levels < 1.04 mmol/L (40 mg/dL), triglyceride levels ≥ 1.70 mmol/L (150 mg/dL), or prescribed lipid-lowering medications. Cardiovascular disease at baseline was defined as any self-reported history of coronary heart disease, stroke, or transient ischemic attack.

### Statistical analysis

Data are expressed as mean ± standard deviation [SD] for normally distributed variables. The differences in values between baseline characteristics were assessed using ANOVA for continuous variables and the Pearson’s χ2 test for categorical variables. Restrictive cubic splines were used to visualize the relationship rAIp75 and RFD, and then logistic regression analysis were performed to observe the effects of baseline rAIp75 on RFD after adjustment for age, sex, BMI, baseline eGFR, current smoking status, current drinking status, medical history (hypertension) as well as the usage of antihypertensive, hypoglycemic, and lipid-lowering drugs. Subgroup and interaction analysis was performed with regard to sex. A *P* value of < 0.05 (two-sided) was considered statistically significant for all tests. All analyses were performed using Empower(R) (www.empowerstats.com, X&Y Solutions, Inc. Boston MA) and R (http://www.R-project.org).

## Results

Table 1 shows the characteristics of the participants overall and stratified by tertiles of baseline rAIp75. The mean (SD) age of the participants was 56.64 ± 8.48 years, and 64.01% (2026/3165) of the participants were female. The prevalence of hypertension and diabetes mellitus was 49.23% (1558/3165) and 23.54% (745/3165), respectively. rAIp75 was 80.49 ± 11.96%. The baseline eGFR was 101.18 ± 10.63 mL/min/1.73 m^2^ and the follow-up eGFR was 98.36 ± 11.91 mL/min/1.73 m^2^. Participants with higher rAIp75 were more likely to be female and had a significantly lower BMI, pulse, and prevalence of current smoking and drinking. Neither the prevalence of hypertension, diabetes mellitus, dyslipidemia, or cardiovascular disease nor the usage of antihypertensive, hypoglycemic, or lipid-lowering drugs significantly differed between participants with different rAIp75 levels.

**Table 1:** Characteristics of participants stratified by baseline rAIp75

Variables	Total	rAIp75, %			*p*
		Tertile 1 (37–75)	Tertile 2 (76–84)	Tertile 3 (85–142)	
*N*	3165	1021	1020	1124	
Age, years	56.64 ± 8.48	55.85 ± 8.18	57.39 ± 8.63	56.67 ± 8.53	< 0.001
Female, *n* (%)	2026 (64.01%)	396 (38.79%)	698 (68.43%)	932 (82.92%)	< 0.001
BMI, kg/m^2^	26.03 ± 3.36	26.30 ± 3.35	26.04 ± 3.46	25.77 ± 3.25	0.001
Pulse, beats/min	76.15 ± 11.75	77.37 ± 12.41	75.76 ± 11.31	75.40 ± 11.44	< 0.001
rAIp75, %	80.49 ± 11.96	67.51 ± 6.87	80.17 ± 2.55	92.56 ± 7.40	< 0.001
Baseline eGFR, mL/min/1.73m^2^	101.18 ± 10.63	101.21 ± 10.98	100.50 ± 10.52	101.77 ± 10.39	0.023
Follow-up eGFR, mL/min/1.73m^2^	98.36 ± 11.91	99.03 ± 12.55	97.80 ± 11.06	98.25 ± 12.04	0.062
Current smoking, *n* (%)	588 (18.58%)	292 (28.60%)	156 (15.29%)	140 (12.46%)	< 0.001
Current alcohol intake, *n* (%)	729 (23.03%)	361 (35.36%)	195 (19.12%)	173 (15.39%)	< 0.001
Prevalence of disease					
Hypertension, *n* (%)	1558 (49.23%)	517 (50.64%)	510 (50.00%)	531 (47.24%)	0.243
Diabetes mellitus, *n* (%)	745 (23.54%)	244 (23.90%)	235 (23.04%)	266 (23.67%)	0.894
Dyslipidemia, *n* (%)	2256(71.28%)	727 (71.20%)	702 (68.82%)	827 (73.58%)	0.052
Self-reported cardiovascular disease, *n* (%)	391(12.35%)	138(13.52%)	122(11.96%)	131(11.65%)	0.382
Medication					
Antihypertensive medications	982(31.20%)	329 (32.29%)	324 (32.14%)	329 (29.38%)	0.257
Hypoglycemic medications	310(9.83%)	96 (9.42%)	108 (10.66%)	106 (9.44%)	0.555
Lipid-lowering medications	327(10.44%)	104 (10.27%)	116 (11.54%)	107 (9.60%)	0.335

*BMI* body mass index, *eGFR* estimated glomerular filtration rate, *rAIp75* radial augmentation index per 75 heart beats

**Fig. 1:**
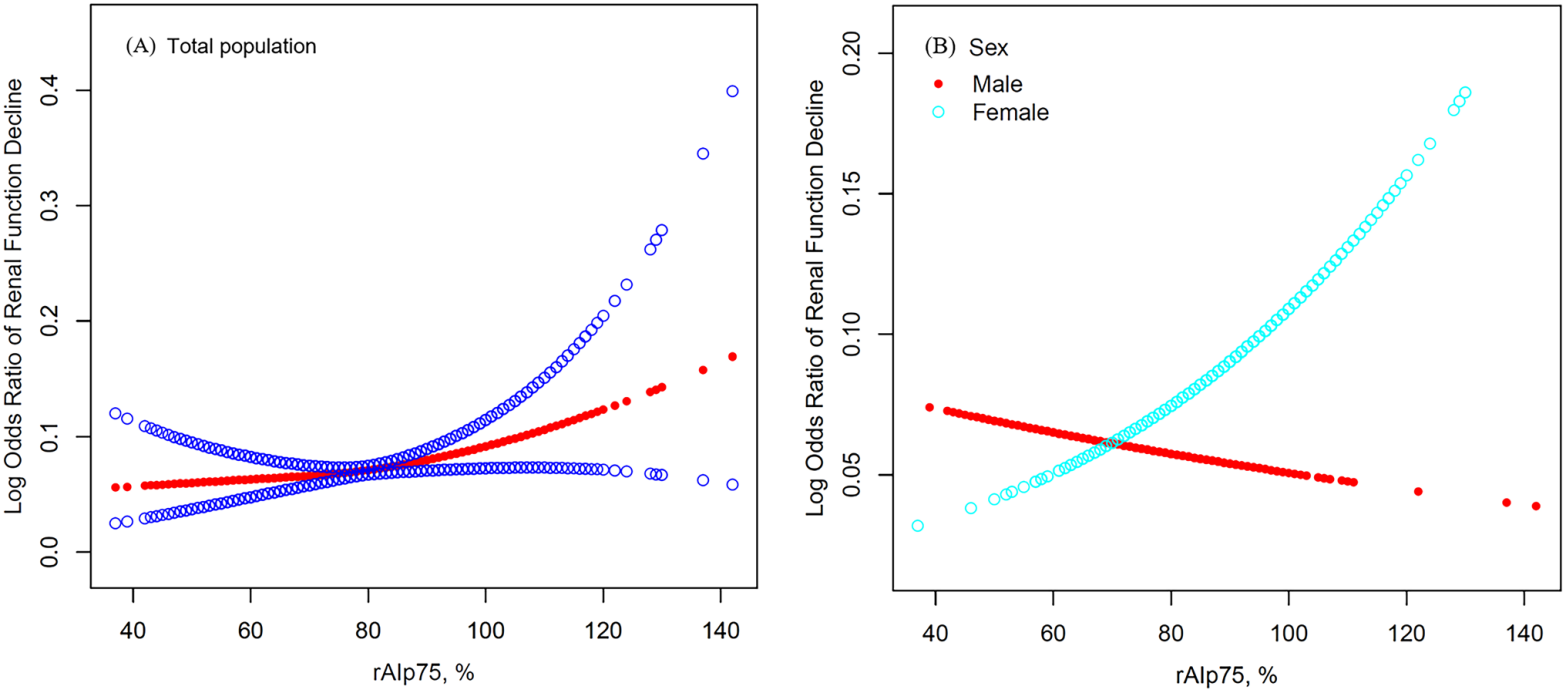
Smooth curve fitting of the rAIp75 and RFD in total population (**a**) and in subgroup stratified by sex (**b**). Variables in the model: age, sex, body mass index, baseline eGFR, current smoking status, current drinking status, hypertension, diabetes mellitus, dyslipidemia, self-reported cardiovascular disease, antihypertensive medications, hypoglycemic medications, and lipid-lowering medications. *rAIp75* radial augmentation index per 75 heart beats

After a mean 2.35-year follow-up, the incidence of RFD was 7.30%. Smooth curve fitting (Fig. 1a) and logistic regression analysis (Table 2) were conducted to assess the association between the rAIp75 and RFD. The smooth curve fitting presented that elevated rAI was related to the increased prevalence of RFD in general population. In the crude models, rAIp75 was significantly associated with RFD (odds ratio [OR] = 1.17, 95% confidence interval [CI]: 1.05–1.31, *p* = 0.005). However, the association did not persist after adjustment for possible confounders (OR = 1.12, 95% CI: 0.99–1.27, *p* = 0.074).

**Table 2:** Multivariate regression analysis of the effect of the rAIp75 on RFD

Variable	Crude model (*N* = 3165)		Adjusted model 1 (*N* = 3165)		Adjusted model 2 (*N* = 3121)	
	OR (95% CI)	*p*	OR (95% CI)	*p*	OR (95% CI)	*p*
rAIp75, per 10% increase	1.17 (1.05, 1.31)	0.005	1.12 (1.00, 1.27)	0.058	1.12 (0.99, 1.27)	0.074
rAIp75 categories						
T1 (37–75)	1		1		1	
T2 (76–84)	1.05 (0.74, 1.50)	0.782	0.94 (0.65, 1.36)	0.737	0.94 (0.65, 1.37)	0.745
T3 (85–142)	1.46 (1.05, 2.02)	0.023	1.26 (0.89, 1.80)	0.195	1.26 (0.88, 1.81)	0.204
*p* for trend		0.018		0.144		0.154

Adjusted Model 1: adjusted for age and sex

Adjusted Model 2: adjusted for: age, sex, body mass index, baseline estimated glomerular filtration rate, current smoking status, current drinking status, hypertension, diabetes mellitus, dyslipidemia, self-reported cardiovascular disease, antihypertensive medications, hypoglycemic medications, and lipid-lowering medications

*OR* odds ratio, rAIp75 radial augmentation index per 75 heart beats, *RFD* renal function decline, *CI* confidence interval

As shown in Table 3, the effect of baseline rAIp75 on RFD was modified by sex (p for interaction = 0.038). The relationship between rAIp75 and RFD was observed in women (OR = 1.23, 95% CI: 1.06–1.43, *p* = 0.007), but not in men (OR = 0.94, 95% CI: 0.76–1.16, *p* = 0.542). The trends in the two subgroups shown by smooth curves in Fig. 1b were consistent with the analysis above.

**Table 3:** Interactive effect between sex and the rAIp75 on RFD

Sex	RFD, *n*(%)	OR (95% CI)	*p*	*p* for interaction
Male	67(5.9%)	0.94 (0.76, 1.16)	0.5420	**0.038**
Female	164(8.1%)	1.23 (1.06, 1.43)	0.0074	

Variables in the model: age, sex, body mass index, baseline eGFR, current smoking status, current drinking status, hypertension, diabetes mellitus, dyslipidemia, self-reported cardiovascular disease, antihypertensive medications, hypoglycemic medications, and lipid-lowering medications

*CI* confidence interval, *OR* odds ratio, *rAIp75* radial augmentation index per 75 heart beats, *RFD* renal function decline

## Discussion

In this Chinese community-based study of 3165 participants with an eGFR of ≥ 60 mL/min/1.73 m^2^, the rAI, an indicator of arterial stiffness, was not independently associated with RFD. However, we found that the association between rAI and RFD was modulated by sex. Namely, the rAI was significantly associated with RFD in women, but not in men.

The kidney is a specific high-flow and low-resistance end organ, which renders it more vulnerable than other organs to systemic pulsatile damage in the presence of increased arterial stiffness [6, 22]. Data from Age, Gene/Environment Susceptibility-Reykjavik Study (AGES-RS) included 367 elderly adults with an average GFR of 65 mL/min/1.73 m^2^ and showed an association between arterial stiffness and GFR (slope of regression *β* = − 2.28 ± 0.85 mL/min per SD, *p* = 0.008); however, the relation was no longer significant (*p* = 0.10) after taking into account the pulsatility index. Mediation analysis revealed that 34% of the association between aortic stiffness and GFR was mediated by the pulsatility index (95% CI of indirect effect: 21.35–20.29), indicating that the relation between aortic stiffness and lower GFR may be mediated by the transmission of excessive pulsatility to the renal microvasculature [6]. The rAI, derived from the pressure waveform resulting from applanation tonometry that applies slight pressure on the radial artery, is considered a useful and easily obtainable marker of arterial stiffness [17, 23]. Studies have demonstrated that the rAI is associated with a rapid deterioration of renal function in patients with moderately to severely decreased renal function [10, 24, 25]. A study by Weber et al., including patients with stage 3–4 CKD, demonstrated that each 10% increase in rAI was associated with a 47% increased risk of reaching the combined renal endpoint of doubling serum creatinine or of requiring dialysis or transplantation (HR = 1.474, 95% CI: 1.020–2.030) [10]. Similarly, Taal et al. [25] reported that the rAI was an independent risk factor for end-stage renal disease in patients with CKD stages 4–5 (HR = 1.08, 95% CI: 1.04–1.14).

However, to the best of our knowledge, only two prospective studies have examined the relationship between the rAI and RFD in patients with preserved renal function [26, 27]. Data on a multiethnic cohort including 5232 participants in the US showed that the rAI by tertile was not related to rapid RFD (defined as an annual eGFR loss of > 3 mL/min/1.73 m^2^) during a 5-year period (OR = 0.98, 95% CI: 0.82–1.16)[26]. Similarly, a longitudinal analysis from a community-based cohort of 948 participants that were followed up for 5 years in China revealed that the rAI had no statistically independent association with any rapid RFD (defined as an annual GFR loss of > 3 mL/min/1.73 m^2^) (HR = 0.994, 95% CI: 0.974–1.015) [27]. The present study is by far the largest study in China to explore the relationship between the rAI and RFD in a non-CKD population. Unlike previous studies, RFD was defined by the standard of the Kidney Disease: Improving Global Outcome in this study [21]. More importantly, although we did not find an independent correlation between the rAI and RFD in the overall population, further subgroup analysis revealed this correlation in women group. This finding suggests that female may be a factor that enhances the effect of rAI on RFD. However, it is still unclear how sex modifies the relationship between rAI and RFD, and thus further studies are needed.

Several limitations to our study should be acknowledged. First, the sample size of our study was relatively small, and the follow-up period was not long enough. Second, serum creatinine at baseline and follow-up was measured in different methods in different laboratories, which may lead to some deviations. However, these creatinine results were transformed into values measured by enzymatic method and calibrated to the values of the third laboratory. Third, the eGFR decline endpoints were based on measurements acquired at only two time points; this most likely reduces the accuracy of our assessment. Fourth, participants in this Beijing community-based study were recruited by responding to recruitment posters or invited phone calls instead of using random sampling method; this might contribute to sampling bias to some extent. Finally, only Chinese participants were included in our study, which limits the generalizability of our findings to other populations.

## Conclusions

This study, conducted on a Chinese community-based population with an eGFR of ≥ 60 mL/min/m^2^, identified an association between increased arterial stiffness and RFD in women. The rAI might represent a suitable measurement for large-scale populations and may facilitate the identification of individuals at high risk of RFD.
